# A Bioinformatic Approach for the Identification of Molecular Determinants of Resistance/Sensitivity to Cancer Thermotherapy

**DOI:** 10.1155/2019/4606219

**Published:** 2019-11-11

**Authors:** Mustafa Barbaros Düzgün, Konstantinos Theofilatos, Alexandros G. Georgakilas, Athanasia Pavlopoulou

**Affiliations:** ^1^Izmir Biomedicine and Genome Center (IBG), 35340 Balcova, Izmir, Turkey; ^2^Izmir International Biomedicine and Genome Institute, Dokuz Eylül University, 35340 Balcova, Izmir, Turkey; ^3^InSyBio Ltd, Innovations House, 19 Staple Gardens, Winchester, SO23 8SR, UK; ^4^DNA Damage Laboratory, Physics Department, School of Applied Mathematical and Physical Sciences, National Technical University of Athens, Iroon Polytechniou 9, 15780 Zografou, Greece

## Abstract

Application of heat above 43°C and up to 47°C, the so-called “thermal ablation” range, leads to tumor cell destruction either by apoptosis or by necrosis. However, tumor cells have developed mechanisms of defense that render them thermoresistant. Of importance, the *in situ* application of heat for the treatment of localized solid tumors can also prime specific antitumor immunity. Herein, a bioinformatic approach was employed for the identification of molecular determinants implicated in thermoresistance and immunogenic cell death (ICD). To this end, both literature-derived (text mining) and microarray gene expression profile data were processed, followed by functional enrichment analysis. Two important functional gene modules were detected in hyperthermia resistance and ICD, the former including members of the heat shock protein (HSP) family of molecular chaperones and the latter including immune-related molecules, respectively. Of note, the molecules HSP90AA1 and HSPA4 were found common between thermoresistance and damage signaling molecules (damage-associated molecular patterns (DAMPs)) and ICD. In addition, the prognostic potential of *HSP90AA1* and *HSPA4* overexpression for cancer patients' overall survival was investigated. The results of this study could constitute the basis for the strategic development of more efficient and personalized therapeutic strategies against cancer by means of thermotherapy, by taking into consideration the genetic profile of each patient.

## 1. Introduction

Cancer is a debilitating disease with a high mortality rate and increasing prevalence [1]. The current widely used therapeutic strategies against cancer include chemotherapy and radiotherapy, either alone or combined [2]. Despite the advancements in radiotherapy techniques and the discovery of potent chemotherapeutic agents, a more effective therapeutic strategy is required to minimize the adverse effects of the current modalities and improve patients' overall survival [3–5]. Thermotherapy represents a revolutionary alternative approach to cancer treatment, based upon the principle that cancer cells exhibit relatively higher sensitivity to increased temperature compared to normal cells [6, 7]. In a seminal study by Dewey et al., it was suggested that the radioresistant cancer cell populations in S-phase or in hypoxic milieu are highly sensitive to elevated temperatures [8]. From a physiological perspective, hyperthermia treatment eliminates oxygen-deprived and usually radioresistant tumor cells by virtue of improved perfusion along with increased blood flow to the tumor site. In this way, not only oxygen concentration is restored in solid tumors but also drug efficacy is improved significantly [9–11].

Cancer cell death can occur in an immunological or nonimmunological fashion. A large number of human cells are eliminated constantly through programmed cell death (PCD) without inducing local or systematic inflammation. Tumor cells undergoing “classical apoptosis” exhibit a tolerogenic or “silent” phenotype. However, certain types of cytotoxic anticancer drugs, as well as radiotherapy and heat treatment, have been demonstrated to induce immunogenic cell death (ICD) [12–17]. The immunostimulatory effect of ICD depends on the emission of certain intracellular factors to the extracellular milieu, referred to as damage-associated molecular patterns (DAMPs). DAMPs are endogenous danger signaling molecules, including heat shock proteins (HSPs), high-mobility group box 1 (HMGB1), S100 proteins, calreticulin, DNA, RNA, reactive oxygen species (ROS), and adenosine triphosphate (ATP) [12, 18–26]. These molecules have the capacity to elicit systemic responses via immune pathways associated with antigen-presenting cell (APC) maturation/activation and antigen processing/presentation [18, 27–30]. DAMP release has been found to be implicated in cell death mechanisms that contribute to immunostimulatory processes such as pyroptosis and pyronecrosis; nevertheless, necrosis, the “accidental cell death,” has been long known to be associated with DAMPs [12, 31]. Depending on tissue type, heat above 43°C, the so-called “thermal ablation range,” leads to tumor cell destruction predominantly by necrosis, whilst 41-43°C promotes cell death mainly by apoptosis [32]. Hyperthermia has the capacity to induce cytotoxicity in cancer cells and prime both innate and adaptive immunity [33, 34].

Of importance, there is evidence to suggest a critical role of oxidative stress in thermo-induced cytotoxicity [35, 36]. Heat increases the cellular generation of reactive oxygen species (ROS), including hydrogen peroxides, hydroxyl radicals, and superoxide anion, thereby resulting in damage to DNA, proteins, and lipid membranes. The rapid production of ROS, following thermotherapy, surpasses the ability of cellular antioxidant enzymes, such as catalase, superoxide dismutase, and glutathione peroxidase, to detoxify ROS effectively, leading eventually to cell death [35, 36].

The development of resistance of cancer cells to chemoradiotherapy, mainly due to intrinsic and acquired factors, represents a major limitation in the treatment of a variety of cancer types [37–39]. Likewise, thermotherapy also results in the development of resistance in cancerous cells [40, 41]. Upon heat-induced stress, HSPs are activated as a universal response to protect the proteome of the cell [42, 43]. In particular, several studies have demonstrated that Hsp27, Hsp70, and Hsp90 play a pivotal role in conferring tolerance against hyperthermia treatment [44–46]. Accordingly, a number of HSP family members, which also function as molecular chaperones, are implicated in cytoprotective pathways that regulate proteome integrity, protein homeostasis (proteostasis), apoptosis, cellular proliferation, and senescence. Chaperones exert holdase and foldase activities to prevent off-pathway protein folding trajectories that produce nonnative protein conformations and aggregation, whilst favoring the native conformation of proteins [47–49]. In eukaryotes, stress-induced transcription of *HSP* genes is regulated by the heat shock factor 1 (HSF1), referred to as the “master regulator of heat shock response” [50]. HSF1 has been shown to be associated with tolerance against lethal temperatures (45°C for 60 minutes) following conditioning heat treatment (43°C for 30 minutes) in mouse embryonic fibroblasts (MEFs) [51].

Elucidation of the underlying mechanisms of resistance to heat-induced stress and ICD is of paramount importance in improving the clinical efficacy of anticancer heat therapy and customize it to the individual patient. Herein, we have made an effort to unravel the molecular determinants and the corresponding pathways implicated in thermoresistance/ICD in cancer cells by employing both text mining and bioinformatic approaches.

## 2. Methods

### 2.1. Bibliographic Search

Manual text mining approaches were employed for extracting gene terms related to “thermotherapy”, “heat therapy”, “resistance”, “sensitivity”, “cancer”, “damage-associated molecular patterns”, and “immunogenic cell death” from the biomedical bibliographic database PubMed/MEDLINE (https://www.ncbi.nlm.nih.gov/pubmed). Collectively, 56 genes (or gene products) were retrieved, for which the official HGNC (HUGO Gene Nomenclature Committee) [52, 53] gene symbols were used.

### 2.2. High-Throughput Gene Expression Data

In addition to systematic literature review, omics data was used in this study. The NCBI GEO (Gene Expression Omnibus) DataSets [54] database was searched extensively using the terms (“heat therapy” or “thermotherapy” or “hyperthermia”) and (“cancer” or “tumor”) and “resistance” and “sensitivity” and (“human” or “homo sapiens”) for gene expression data. In this way, the eligible gene expression microarray GEO Series GSE77310 dataset was obtained, which contains two samples of hyperthermia-resistant (HTR) ovarian cancer cells heat treated at 46°C and two control samples of SKOV3 cells incubated at 37°C. GSE77310 is based on the Illumina HumanHT-12 V4.0 expression beadchip platform (GPL10558).

### 2.3. Differential Gene Expression Analysis

The GEO2R interactive web server [54] was employed to detect differentially expressed genes (DEG) between the HTR and sensitive ovarian cancer cells, by setting absolute log fold changes ∣logFC∣ ≥ 2 and FDR-adjusted *p* value ≤ 0.05. Moreover, GEPIA (Gene Expression Profiling Interactive Analysis) [55], an interactive web-based application for gene expression data analysis of cancer and normal tissues from The Cancer Genome Atlas (TCGA) (https://tcga-data.nci.nih.gov) [56] and the Genotype-Tissue Expression (GTEx) [57, 58] (https://gtexportal.org/home/), was employed to investigate the differential expression patterns of the genes under study.

### 2.4. Pathway Enrichment Analysis

To further explore functional differences between the thermoresistance-associated and the ICD/DAMP genes under investigation, functional enrichment analysis was performed. To this end, WebGestalt (WEB-based GEne SeT AnaLysis Toolkit) [59] was employed to identify statistically significant overrepresented WikiPathways [60] terms within the two gene sets; hypergeometric distribution analysis was used and the threshold for the adjusted *p* value was set at 10^−3^.

### 2.5. Functional Interaction Networks

The associations among the molecules under study were investigated using STRING v11 [61], a database of either known or predicted, direct or indirect, functional associations among proteins and genes. Moreover, Cytoscape v3.7.1 [62], an open source software, was employed for the statistical analysis of networks.

### 2.6. Survival Analysis

The prognostic potential of *HSPA4*and *HSP90AA1*, found to be implicated both in thermoresistance and DAMPs/ICD, for several types of cancers was investigated. The relationship between *HSPA4* and *HSP90AA1* overexpression and cancer patients' overall survival (OS) was explored through SurvExpress [63], an online tool for biomarker validation; the datasets for survival analysis were acquired from TCGA [56].

### 2.7. Gene Expression Correlation Analysis

Gene correlation analyses based on mRNA expression levels were performed using GEPIA [55] which analyzes RNA sequencing (RNA-Seq) expression data from TCGA [56].

### 2.8. Melting Temperature Estimation

The SeqUtils package of Biopython version 1.73 [64] was used to estimate the melting temperature (*T*_m_) of thermoresistant and DAMP/ICD proteins. Specifically, the method described by Ku et al. [65] was used to estimate the temperature at which 50% of the protein is unfolded, directly from protein sequences.

## 3. Results

### 3.1. Identification of Thermoresistance and DAMP/ICD-Associated Molecules

**Figure 1:**
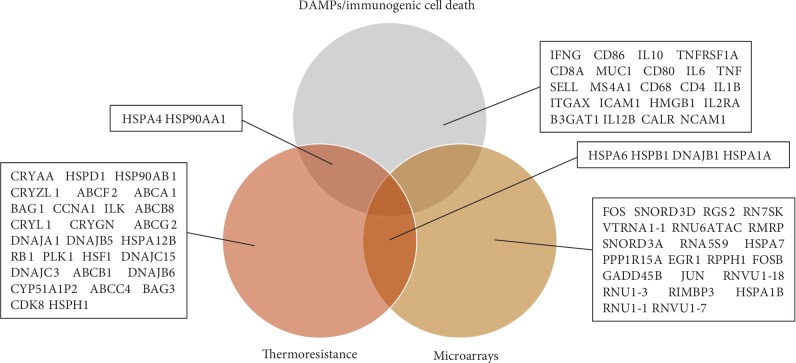
Venn diagram of the thermoresistance and DAMP/ICD-associated molecules.

The genes/gene products detected through extensive literature text mining are listed in [Supplementary-material supplementary-material-1]. From the omics data (thermomics), a total of 26 genes were found to be differentially expressed between the heat-resistant and heat-sensitive ovarian cancer cells by analyzing GSE77310, all of them upregulated, suggesting that it is indispensable for cells to respond to thermal stress. A Venn diagram depicting the genes/gene products associated with thermoresistance and DAMP/ICD was created using BioVenn [66] (Figure 1). A total of 56 thermoresistance-associated microarray-derived genes and literature-extracted genes/gene products and 24 DAMP genes/proteins were detected. Among the literature-derived genes, hereafter referred to as “thermogenes,” the evolutionarily highly conserved Hsp70 is the most prominent family with four distinct homologs, namely, HSPA12B, HSPA1A, HSPA4, and HSPA6 ([Supplementary-material supplementary-material-1]). Of note, the molecules HSP90AA1 and HSPA4 were found common between thermoresistance and DAMPs/ICD.

### 3.2. WikiPathways Enrichment Analysis

Based on WikiPathways enrichment analysis, immune-related pathways, such as inflammatory response pathway, TNF alpha signaling pathway, Th1-Th2, and cytokines and inflammatory response, were significantly enriched within the DAMP/ICD gene set (Table 1). Moreover, several cancer-related pathways including prostate cancer, integrated pancreatic cancer pathway, and oncostatin M signaling pathway were particularly overrepresented in thermoresistance-associated genes (Table 1). Overall, significant functional differences between the molecular determinants of thermoresistance and DAMPs/ICD were found. Thus, these molecules could serve as possible diagnostic signatures for cancer patients' response to hyperthermia treatment.

### 3.3. Estimated Melting Temperature

In order to gain a better mechanistic understanding of the role of the proteins encoded by the retrieved thermogenes, the average melting temperature was calculated for the thermoresistant and DAMP/ICD proteins collectively (Table 2) and the individual proteins ([Supplementary-material supplementary-material-1]). The average *T*_m_ was higher for the thermoresistance-relevant proteins (67°C) as compared to the DAMP/ICD proteins (63.42°C) (Table 2). Interestingly, DNAJB5 was found to have the highest estimated melting temperature (84°C) ([Supplementary-material supplementary-material-1]), suggesting that this protein is extraresistant to heat-induced stress.

### 3.4. Network Analysis

**Figure 2:**
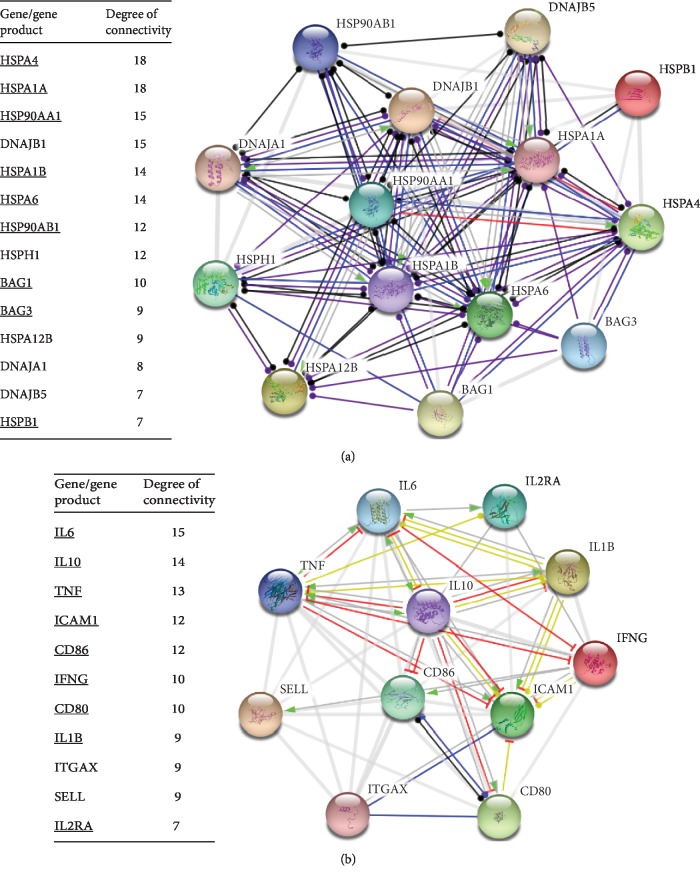
Network modules of the most highly connected genes related to (a) thermoresistance and (b) DAMPs/ICD. The nodes represent genes/proteins and the connecting lines (edges) functional links, respectively. The edges connecting the nodes indicate the mode of action of the interacting molecules with a confidence score above 0.7. The molecules implicated in the relevant WikiPathways are underlined.

As it is shown in [Supplementary-material supplementary-material-1], 27 out of 56 gene/gene products implicated in thermoresistance form a highly interconnected network with a significant confidence. Likewise, 23 out of 24 genes/proteins related to DAMPs/ICD are also interconnected ([Supplementary-material supplementary-material-1]). Of note, HSPA4 and HSP90AA1 appear to have very few links in the “DAMP/ICD” network, whereas in the “thermoresistance” networks they have many links to their neighboring nodes ([Supplementary-material supplementary-material-1]). This further supports the “bystander effect” of HSPs on ICD, that is, the rather limited role of HSPs in ICD. Moreover, networks of the DAMP/ICD and thermoresistance-associated gene/gene products with the highest degree of connectivity (i.e., the highest number of links to the neighboring nodes) in the original networks, shown in [Supplementary-material supplementary-material-1], were generated (Figure 2). These highly connected genes appear to be also interconnected in corresponding dense networks with a confidence score above 0.7 (Figure 2).

### 3.5. Expression Profiling of Highly Connected Genes

**Figure 3:**
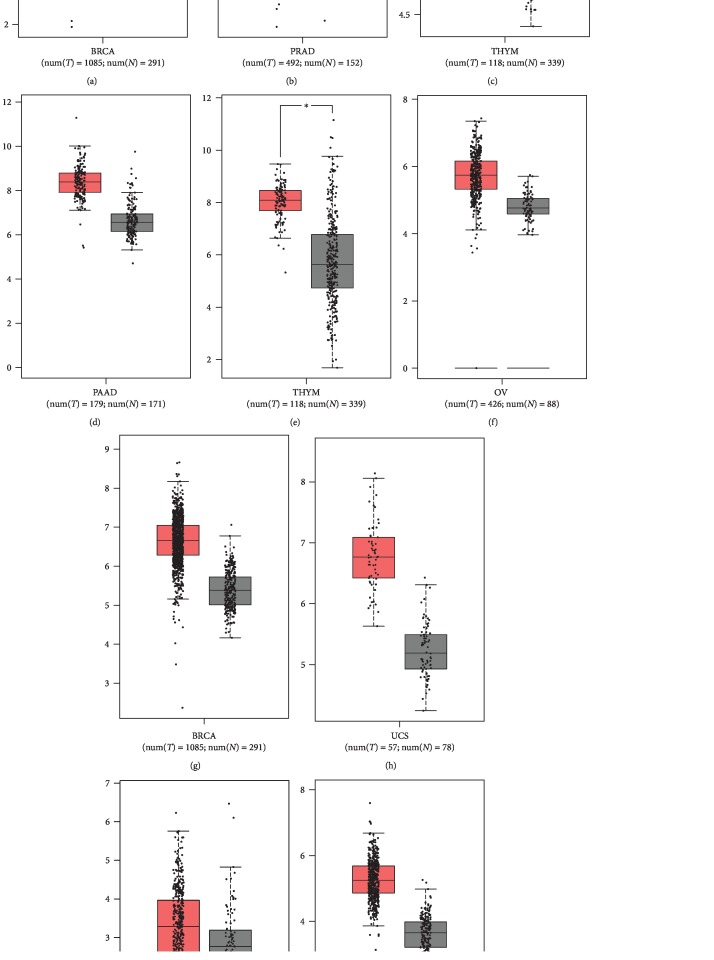
Differential expression of (a) *HSPA1A*, (b) *HSP90AB1*, (c) *BAG1*, (d, e) *HSP90AA1*, (f–h) *HSPA4*, and (i, j) *DNAJB5* in different cancers. BRCA: breast invasive carcinoma; LGG: brain lower grade glioma; OV: ovarian serous cystadenocarcinoma; PAAD: pancreatic adenocarcinoma; PRAD: prostate adenocarcinoma; THYM: thymoma; UCS: uterine carcinosarcoma.

The differential expression profiles of six of the most highly connected thermogenes shown in Figure 2 were investigated in different types of cancers (Figure 3). The six thermogenes include *HSP90AA1* and *HSPA4*, common in thermoresistance and DAMPs/ICD, *DNAJB5*, the protein product of which has the highest estimated thermostability ([Supplementary-material supplementary-material-1]), and *HSPA1A*, *HSP90AB1*, and *BAG1*, which are implicated in cancer-relevant pathways (Figure 2(a), underlined). All six thermogenes appear to be significantly overexpressed in breast cancer (*HSPA1A*, *HSPA4*), gliomas (*DNAJB5*), ovarian cancers (*HSPA4*, *DNAJB5*), pancreatic cancers (*HSP90AA1*), prostate cancer (*HSP90AB1*), thymic carcinoma (*BAG1*, *HSP90AA1*), and uterine cancers (*HSPA4*), as compared to normal tissue (Figure 3).

### 3.6. HSP90AA and HSPA4 Are Potential Prognostic Markers for Diverse Cancer Types

**Figure 4:**
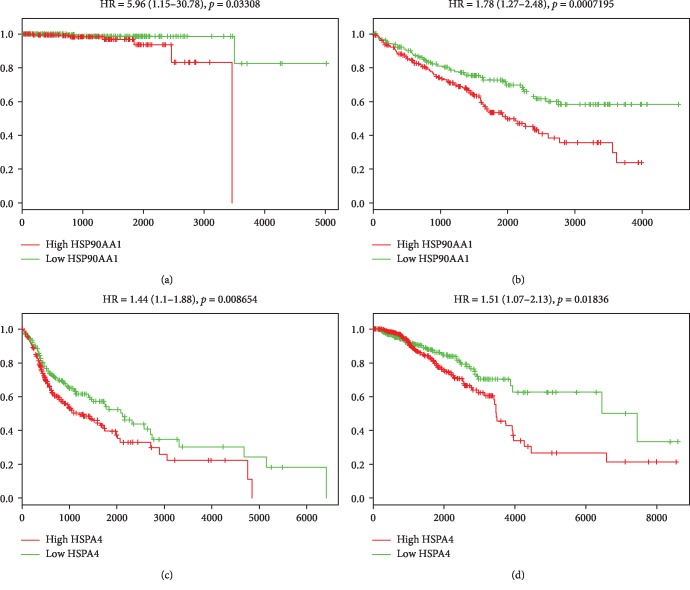
Survival graphs representing the prognostic potential of *HSP90AA1* for overall survival in (a) prostate adenocarcinoma and (b) kidney renal clear cell and *HSPA4* for OS in (c) head and neck squamous cell carcinoma and (d) breast invasive carcinoma. The HRs with the corresponding 95% confidence interval values (within parentheses) and *p* values are indicated.

A statistically significant relationship was found between *HSP90AA1* and *HSPA4* overexpression and poor overall survival in cancer patients, as it is indicated by pooled hazard ratio (HR) values greater than 1 and *p* values less than 0.05 (Figure 4). Therefore, *HSP90AA1* and *HSPA4* may have a significant prognostic value for tumors of diverse tissue origin.

## 4. Discussion

In this study, we have made an effort to elucidate the molecular mechanisms of resistance to hyperthermia and the treatment-related ICD by employing a bioinformatic approach. To this end, we identified DEGs associated with thermoresistance, or stress resistance, and DAMPs through text mining and microarray data analysis. In the present study, the thermoresistance-related network module was found to consist exclusively of HSPs. This finding is consistent with the fact that HSPs constitute major components of a cell and they play a vital role in protein folding, activity, turnover, and trafficking. Thus, they can counteract cellular stress through their intrinsic chaperoning activity [48]. We suggest that the HSP module maintains proteostasis through enhanced preservation of the structural integrity of proteins essential to stress tolerance, including oncogenic proteins. Accordingly, many HSPs were shown to dysregulate programmed cell death and proliferation by stabilizing mutant forms of tumor suppressor proteins like p53 and MSH2 (actively involved also in DNA repair), as well as overexpressed oncogenic proteins [67] such as PLK1 [68]. In our study, several key genes implicated in thermoresistance were found to be markedly overexpressed in tumors of different tissue origin (Figure 3). Moreover, the expression patterns of the thermogenes *HSPA4* and *HSP90AA1* and the oncogene *PLK1* were found to be positively correlated in diverse types of cancers ([Supplementary-material supplementary-material-1]). In this context, it is plausible to suggest that HSPs can greatly contribute to oncogenesis under hyperthermic stress.

Besides, there is concrete evidence that high expression of HSPs is associated with diverse types of cancers and negative prognosis in the clinical outcomes of cancer patients [69]. Members of the Hsp90 and Hsp70 family could serve as predictors for worse prognosis in cancer patients, since overexpression of *HSP90AA1* and *HSPA4*, respectively, was shown to be associated with worse overall survival in different types of cancers (Figure 4).

Notably, HSPs also contribute to the proper folding of ROS-detoxifying enzymes under proteotoxic stress. For example, Hsp70 was shown to increase the activity of the glutathione peroxidase and glutathione reductase under stress [70]. Also, we found that the expression levels of *HSPA4* and *HSP90AA1* and the detox enzyme gene *CAT* (*catalase*) are positively correlated in cancer ([Supplementary-material supplementary-material-1]). However, the rapid production of ROS overwhelms the activity of the detox enzymes to remove ROS, therefore rendering cancer cells more sensitive upon heat stress [36]. Of interest, no ROS-detoxifying enzyme genes were found to be overexpressed through microarray-based expression analysis, further supporting the limited activity of detox enzymes under thermal stress (Figure 1).

Of interest, no cellular compartment-specific homologs of HSP70 such as HSPA5 (i.e., a binding immunoglobulin protein (BiP) localized in the endoplasmic reticulum) and HSPA9 (i.e., a mortalin localized in the mitochondria) were detected in this study, leading to the suggestion that (i) in the thermotolerant, or oxidative stress tolerant, cancer cells, maintenance of the structural integrity of the respective clients of HSP70s is not obligatory or (ii) HSPA4, which is located in multiple cellular compartments, can likely substitute for the protein folding activity of HSPA5/9. Moreover, *HSPA6* was shown to be upregulated both in heat-treated serous ovarian carcinoma cell lines (Figure 1) and ovarian clear-cell adenocarcinoma by Court et al. [71]. Thus, we could suggest that cancers originating from different tissues might require same HSP70 homologs in stress resistance. Moreover, the *Hsp70* homolog, *HSPA1A*, was found both through microarray analysis and text mining, in this study, to be overexpressed in ovarian cancer, as well as lung carcinoma according to a previous study [72]. Therefore, we could suggest that distinct types of cancers might require identical HSP70 chaperone functions in order to obtain thermoresistance.

DnaJ/Hsp40 family members serve as cochaperones of Hsp70 by playing a role in substrate recruitment and the maintenance of the ATPase cycle of HSP70 [73]. In our study, we detected six members of the DnaJ/Hsp40 family, indicating that thermoresistance entails proteomic stabilization via a Hsp70-independent holdase activity of multiple Hsp40s and/or by contributing to the regulation of Hsp70. Moreover, in our study, HSP110 and BAG-domain-containing proteins, which function as nucleotide exchange factors of Hsp70 by promoting ADP release, were detected, leading to the suggestion that the intrinsic ATPase activity of Hsp70s is calibrated in thermoresistance [74]. Accordingly, proper adjustment of the HSP70/NEF stoichiometric ratio might play a critical role in thermoresistance, since the transcription of all of the aforementioned chaperones and cochaperones is upregulated significantly.

The canonical chaperone Hsp90 isoforms HSP90AA1 and HSP90AB1 were also found to be upregulated in several cancers including pancreatic adenocarcinoma, thymoma, and prostate adenocarcinoma, respectively (Figure 3). Since Hsp90 plays a vital role in the final conformational maturation of cancer-related proteins, such as kinases and growth factors, upregulation of Hsp90 might result to an enhanced folding capacity of its respective clients to maintain their oncogenic potential in thermoresistance [75, 76]. We have also demonstrated that both HSP90AA1 and HSP90AB1 represent highly connected nodes in the thermoresistance module, suggesting that they play a central role in heat resistance.

HSPA4, HSP90AA1, and calreticulin are the HSPs related to thermoresistance that were also found in the ICD network ([Supplementary-material supplementary-material-1]). This is probably due to the lack of studies regarding other members of the broad HSP70 and HSP90 families, as well as the small HSP family. Of note, calreticulin, a multifunctional chaperone protein, is linked to better prognosis of different types of cancers [77], indicative of its dual role in thermoresistance and ICD. Calreticulin has been also utilized as therapeutic adjuvant in cancer [78]. As anticipated, major proinflammatory cytokines such as IL1B, IL6, IL10, IL12B, IL2RA, TNF, and ING were found in our ICD module (Figure 2(b)). In this module, IL6, IL10, and TNF have a high degree of connectivity, suggesting that these protein molecules might play a pivotal role in the ICD functional network. Of note, HMGB1, a well-known damage signaling molecule, is apparently linked to the central hub of our ICD module; this finding is consistent with previous studies which have demonstrated that HMGB1 stimulates the secretion of IL1B, TNF-*α*, IL6, and IL10 via TLR4 in macrophages [79]. Besides, TNF-*α*, previously shown to promote heat-induced apoptosis, has 13 interactions in the ICD network. Intriguingly, enhanced apoptosis during heat stress was shown to depend on the inhibition of HSF1 by TNF-*α* [80]. Of interest, TNF-*α* superfamily (TNFRS) agonists have emerged as potential cancer treatment adjuvants [81]. For example, hyperthermic perfusion of limbs with melphalane and TNF-*α* has been employed to reduce tumor burden in unresectable limb sarcoma or melanoma [82, 83], perhaps by mitigating thermoresistance through the downregulation of HSF1-regulated HSPs.

Based on our *in silico* calculations, the thermoresistance-relevant proteins were found to exhibit high *T*_m_ values (Table 2), above the thermal ablation temperature (i.e., 43–47°C), consistent with previous circular dichroism experiments [84–86]. This finding was expected since the tertiary structure and activity of the respective proteins must remain unaltered under cellular stress induced by hyperthermia-range temperatures in order to protect the cellular proteome. Interestingly, the average *T*_m_ of DAMPs (63.42°C) is remarkably higher compared to the thermal ablation temperature, highlighting the importance of preserving the three-dimensional protein structures of DAMPs in order to functionally interact with their canonical pattern recognition receptors (PRRs) even under thermal stress.

Taken together, our study represents a comprehensive outlook and analysis of heat-induced stress/oxidative stress and ICD in hyperthermia treatment. Uncovering the associated genes, the underlying mechanisms, and the interplay between these phenomena is of paramount importance in terms of designing therapeutic strategies for the effective sensitization of cancer cells to heat treatment and the concurrent modulation of the immune response fostered by fever-range temperature. Besides, our study could provide the foundation for the rational design of novel drugs that could exclusively target molecular determinants of cancer cell thermoresistance and avoid targeting DAMPs/ICD which promote cancer cell clearance through enhanced immune response.

## Figures and Tables

**Table 1:** Overrepresented WikiPathways of (a) DAMP/ICD and (b) thermoresistance-associated genes.

DAMP/ICD		
WikiPathways	Gene symbol	Adj. *p* value

Cytokines and inflammatory response	IFNG, TNF, IL10, CD4, IL1B, IL6, IL12B	1.29*e*-13
Inflammatory response pathway	IFNG, CD80, IL2RA, CD86, TNFRSF1A	8.18*e*-11
Toll-like receptor signaling pathway	TNF, CD80, IL1B, CD86, IL6, IL12B	3.01*e*-10
Allograft rejection	IFNG, TNF, CD80, IL10, CD86, IL12B	3.01*e*-10
Regulation of toll-like receptor signaling pathway	TNF, CD80, IL1B, CD86, IL6, IL12B	1.15*e*-09
Selenium pathway	IFNG, TNF, IL1B, IL6, ICAM1	1.47*e*-08
TCR signaling pathway	CD8A, CD4, IL1B, IL6	1.06*e*-06
TNF alpha signaling pathway	TNF, HSP90AA1, IL6, TNFRSF1A	1.06*e*-06
Th1-Th2	IL10, IL12B	1.49*e*-05
SIDS susceptibility pathways	TNF, IL10, IL1B, IL6	1.49*e*-05
Alzheimer's disease	TNF, IL1B, TNFRSF1A	2.52*e*-05
Type II interferon signaling (IFNG)	IFNG, IL1B, ICAM1	2.52*e*-05
Senescence and autophagy	IFNG, IL1B, IL6	7.51*e*-05
Monoamine transport	TNF, IL1B	0.0002
NOD pathway	HSP90AA1, IL1B	0.0003
AhR pathway	TNF, HSP90AA1	0.0003
TSLP signaling pathway	IL2RA, IL6	0.0004
TWEAK signaling pathway	TNF, IL6	0.0005
TGF beta signaling pathway	IFNG, TNF	0.0006

Thermoresistance		
WikiPathways	Gene symbol	Adj. *p* value

Parkin-ubiquitin proteasomal system pathway	HSPA4, HSPA1A, HSPA1B, HSPA4	7.81*e*-05
Prostate cancer	JUN, PLK1, ABCC4, FOS, HSP90AB1	0.0001
FAS pathway and stress induction of HSP regulation	JUN, RB1, HSPB1	0.0005
MAPK signaling pathway	JUN, HSPB1, FOS, HSPA1A	0.0005
Integrated pancreatic cancer pathway	JUN, PLK1, EGR1, HSP90AB1	0.0006
TSH signaling pathway	JUN, RB1, FOS	0.0006
Androgen receptor signaling pathway	JUN, RB1, BAG1	0.0009
Oncostatin M signaling pathway	JUN, EGR1, FOS	0.0009
Drug induction of bile acid pathway	ABCC4, ABCB1	0.0009

**Table 2:** Average estimated melting temperature for thermoresistance and DAMP/ICD proteins.

	Thermoresistant proteins	DAMP/ICD proteins
Average melting temperature	67°C	63.42°C
Percentage of denaturated proteins at 45°C	0.00%	0.00%
Percentage of denaturated proteins at 50°C	0.00%	12.5%
Percentage of denaturated proteins at 55°C	3.03%	16.67%
Percentage of denaturated proteins at 60°C	6.06%	33.33%

## Data Availability

The data used to support the findings of this study are available from the corresponding authors upon request.

## Supplementary Materials

Supplementary Materials Figure S1: interaction network of the genes of gene products related to (A) thermoresistance and (B) DAMPs/ICD. The edges connecting the nodes indicate confidence level above 0.7. Figure S2: correlation between (A) HSPA4 and PLK1 expression in head and neck squamous cell carcinoma, lung adenocarcinoma, pancreatic adenocarcinoma, and rectum adenocarcinoma (B) HSP90AA1 and PLK1 expression in head and neck squamous cell carcinoma, lung squamous cell carcinoma, stomach adenocarcinoma, and uterine corpus endometrial carcinoma. (C) HSPA4 and CAT expression in uveal melanoma and uterine carcinosarcoma (D) HSP90AA1 and CAT expression in uveal melanoma and lymphoid neoplasm diffuse large B-cell lymphoma. Table S1: list of biological molecules related to resistance to hyperthermia and DAMPs/ICD; the corresponding gene symbol, gene description, and reference are indicated. Table S2: estimated melting temperature per protein.
