# Supplementary material for: A Bioinformatic Approach for the Identification of Molecular Determinants of Resistance/Sensitivity to Cancer Thermotherapy
Source: Oxid Med Cell Longev. 2019 Nov 11;2019:4606219. doi: 10.1155/2019/4606219 (PMC6878812; doi:10.1155/2019/4606219)
Supplement: Supplementary Materials — Figure S1: interaction network of the genes of gene products related to (A) thermoresistance and (B) DAMPs/ICD. The edges connecting the nodes indicate confidence level above 0.7. Figure S2: correlation between (A) HSPA4 and PLK1 expression in head and neck squamous cell carcinoma, lung adenocarcinoma, pancreatic adenocarcinoma, and rectum adenocarcinoma (B) HSP90AA1 and PLK1 expression in head and neck squamous cell carcinoma, lung squamous cell carcinoma, stomach adenocarcinoma, and uterine corpus endometrial carcinoma. (C) HSPA4 and CAT expression in uveal melanoma and uterine carcinosarcoma (D) HSP90AA1 and CAT expression in uveal melanoma and lymphoid neoplasm diffuse large B-cell lymphoma. Table S1: list of biological molecules related to resistance to hyperthermia and DAMPs/ICD; the corresponding gene symbol, gene description, and reference are indicated. Table S2: estimated melting temperature per protein. [file 4606219.f1.pdf]

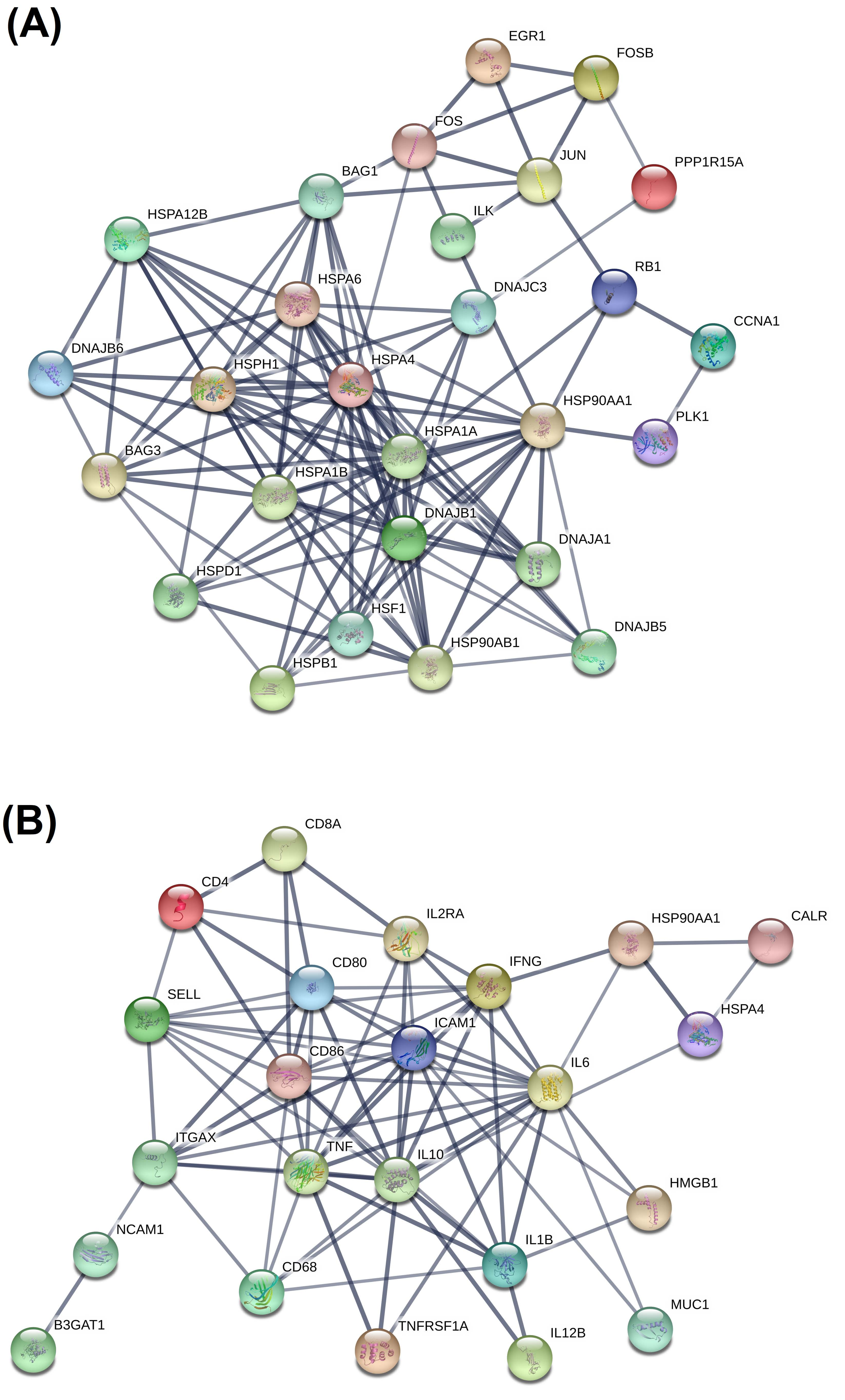
(A)
EGR1
FOSB
FOS
JUN
PPP1R15A
BAG1
HSPA12B
ILK
RB1
HSPA6
DNAJC3
CCNA1
DNAJB6
HSPH1
HSPA4
HSP90AA1
PLK1
HSPA1A
BAG3
HSPA1B
DNAJB1
DNAJA1
HSPD1
HSF1
DNAJB5
HSP90AB1
HSPB1
(B)
CD8A
CD4
IL2RA
HSP90AA1
CALR
CD80
IFNG
SELL
HSPA4
ICAM1
CD86
IL6
ITGAX
TNF
IL10
HMGB1
NCAM1
IL1B
CD68
MUC1
B3GAT1
TNFRSF1A
IL12B

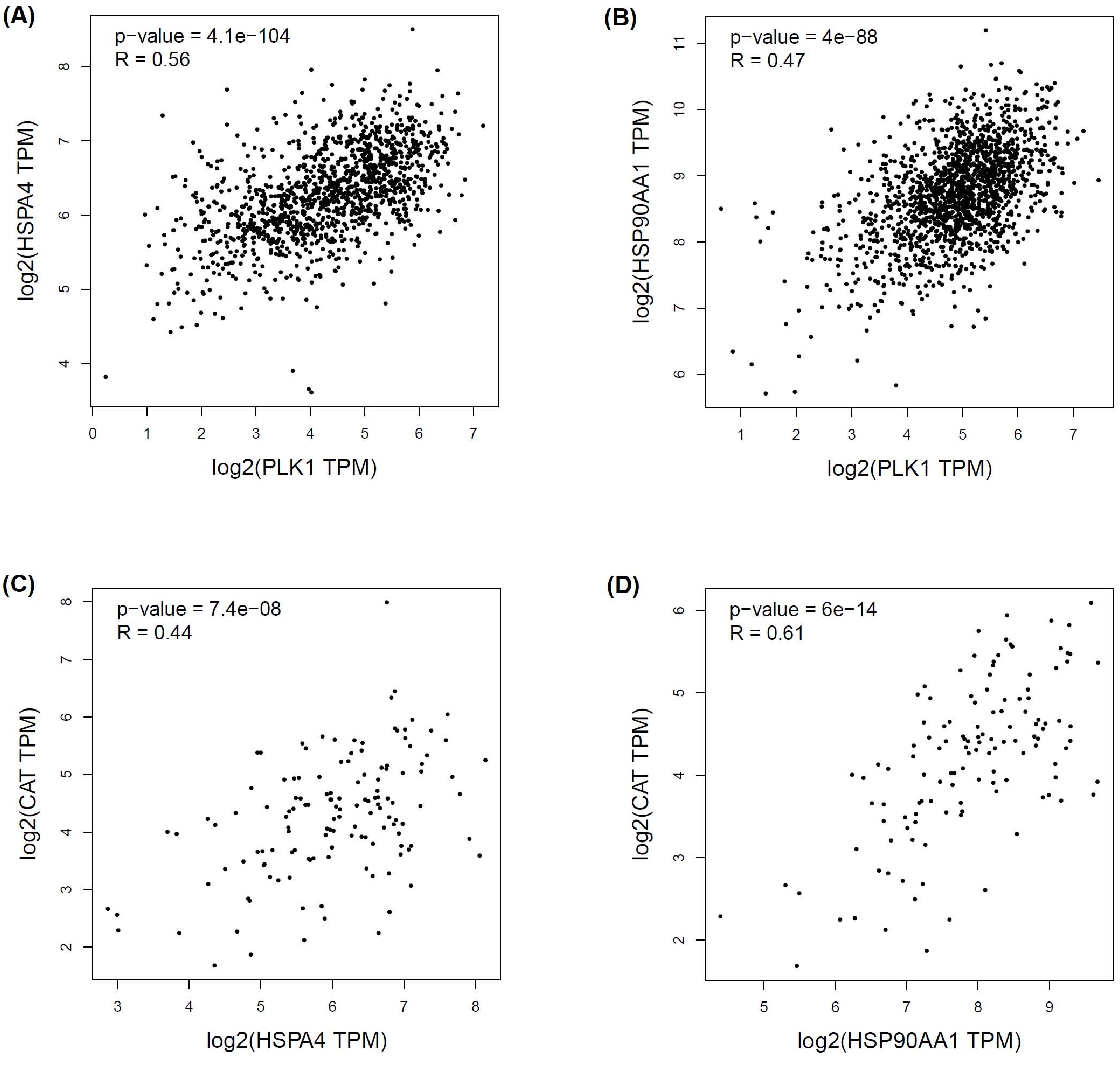

(A)
p−value = 4.1e−104
R = 0.56
log2(HSPA4 TPM)
log2(PLK1 TPM)
(B)
p−value = 4e−88
R = 0.47
log2(HSP90AA1 TPM)
log2(PLK1 TPM)
(C)
p−value = 7.4e−08
R = 0.44
log2(CAT TPM)
log2(HSPA4 TPM)
(D)
p−value = 6e−14
R = 0.61
log2(CAT TPM)
log2(HSP90AA1 TPM)

Thermoresistance		
ABCA1	ATP binding cassette subfamily A member 1	[1]
ABCB1	ATP binding cassette subfamily B member 1	[2]
ABCB8	ATP binding cassette subfamily B member 8	[1]
ABCC4	ATP binding cassette subfamily C member 4	[1]
ABCF2	ATP binding cassette subfamily F member 2	[1]
ABCG2	ATP binding cassette subfamily G member 2 (Junior blood group)	[2]
BAG1	BCL2 associated athanogene 1	[1]
BAG3	BCL2 associated athanogene 3	[3]
CCNA1	cyclin A1	[1]
CDK8	cyclin dependent kinase 8	[1]
CRYAA	crystallin alpha A	[1, 4]
CRYGN	crystallin gamma N	[1]
CRYL1	crystallin lambda 1	[1]
CRYZL1	crystallin zeta like 1	[1]
CYP51A1P2	cytochrome P450 family 51 subfamily A member 1 pseudogene 2	[1]
DNAJA1	DnaJ heat shock protein family (Hsp40) member A1	[1]
DNAJB1	DnaJ heat shock protein family (Hsp40) member B1	[3]
DNAJB5	DnaJ heat shock protein family (Hsp40) member B5	[1]
DNAJB6	DnaJ heat shock protein family (Hsp40) member B6	[1]
DNAJC15	DnaJ heat shock protein family (Hsp40) member C15	[1]
DNAJC3	DnaJ heat shock protein family (Hsp40) member C3	[1]
HSF1	heat shock transcription factor 1	[3]
HSP90AA1	heat shock protein 90 alpha family class A member 1	[5]
HSP90AB1	heat shock protein 90 alpha family class B member 1	[6]
HSPA12B	heat shock protein family A (Hsp70) member 12B	[1]
HSPA1A	heat shock protein family A (Hsp70) member 1A	[7]
HSPA4	heat shock protein family A (Hsp70) member 4	[3, 8]
HSPA6	heat shock protein family A (Hsp70) member 6	[9]
HSPB1	heat shock protein family B (small) member 1	[10]
HSPD1	heat shock protein family D (Hsp60) member 1	[6]
HSPH1	heat shock protein family H (Hsp110) member 1	[11]
ILK	integrin linked kinase	[12]
PLK1	polo like kinase 1	[3]
RB1	RB transcriptional corepressor 1	[1]
DAMPs/Immunogenic cell death		
B3GAT1	beta-1,3-glucuronyltransferase 1	[13]
CALR	calreticulin	[14]
CD4	CD4 molecule	[13, 15-18]
CD68	CD68 molecule	[19]
CD80	CD80 molecule	[20]
CD86	CD86 molecule	[20]
CD8A	CD8a molecule	[13, 15-17]
HMGB1	high mobility group box 1	[14]
HSP90AA1	heat shock protein 90 alpha family class A member 1	[21, 22]
HSPA4	heat shock protein family A (Hsp70) member 4	[21, 22]

ICAM1	intercellular adhesion molecule 1	[23]
IFNG	interferon gamma	[24]
IL1B	interleukin 1 beta	[25]
IL10	interleukin 10	[26]
IL12B	interleukin 12B	[20]
IL2RA	interleukin 2 receptor subunit alpha	[17]
IL6	interleukin 6	[26-28]
ITGAX	integrin subunit alpha X	[29]
MS4A1	membrane spanning 4-domains A1	[13]
MUC1	mucin 1, cell surface associated	[24]
NCAM1	neural cell adhesion molecule 1	[15, 19]
SELL	selectin L	[24]
TNF	tumor necrosis factor	[20, 25]
TNFRSF1A	TNF receptor superfamily member 1A	[28]

References

[1] H. Tonnies, H. Lage, Chromosomal imbalances associated with drug resistance and thermoresistance in human pancreatic carcinoma cells, European journal of cell biology, 83 (2004) 591-601.
[2] U. Stein, H. Lage, A. Jordan, W. Walther, S.E. Bates, T. Litman, P. Hohenberger, M. Dietel, Impact of BCRP/MXR, MRP1 and MDR1/P-Glycoprotein on thermoresistant variants of atypical and classical multidrug resistant cancer cells, International journal of cancer, 97 (2002) 751-760.
[3] T. Yunoki, Y. Tabuchi, A. Hayashi, T. Kondo, Inhibition of polo-like kinase 1 promotes hyperthermia sensitivity via inactivation of heat shock transcription factor 1 in human retinoblastoma cells, Investigative ophthalmology & visual science, 54 (2013) 8353-8363.
[4] I.P.R. van den, P. Overkamp, U. Knauf, M. Gaestel, W.W. de Jong, Alpha A-crystallin confers cellular thermoresistance, FEBS letters, 355 (1994) 54-56.
[5] R. Yang, Q. Tang, F. Miao, Y. An, M. Li, Y. Han, X. Wang, J. Wang, P. Liu, R. Chen, Inhibition of heat-shock protein 90 sensitizes liver cancer stem-like cells to magnetic hyperthermia and enhances anti-tumor effect on hepatocellular carcinoma-burdened nude mice, International journal of nanomedicine, 10 (2015) 7345-7358.
[6] G. van Dongen, W.L. Geilenkirchen, J. van Rijn, R. van Wijk, Increase of thermoresistance after growth stimulation of resting Reuber H35 hepatoma cells. Alteration of nuclear characteristics, non-histone chromosomal protein phosphorylation and basal heat shock protein synthesis, Experimental cell research, 166 (1986) 427-441.
[7] C. Botzler, R. Issels, G. Multhoff, Heat-shock protein 72 cell-surface expression on human lung carcinoma cells in associated with an increased sensitivity to lysis mediated by adherent natural killer cells, Cancer immunology, immunotherapy : CII, 43 (1996) 226-230.
[8] Z. Wang, S. Li, M. Zhang, Y. Ma, Y. Liu, W. Gao, J. Zhang, Y. Gu, Laser-Triggered Small Interfering RNA Releasing Gold Nanoshells against Heat Shock Protein for Sensitized Photothermal Therapy, Advanced science, 4 (2017) 1600327.
[9] K.A. Court, H. Hatakeyama, S.Y. Wu, M.S. Lingegowda, C. Rodriguez-Aguayo, G. Lopez-Berestein, L. Ju-Seog, C. Rinaldi, E.J. Juan, A.K. Sood, M. Torres-Lugo, HSP70 inhibition synergistically enhances the effects of magnetic fluid hyperthermia in ovarian cancer, Molecular cancer therapeutics, (2017).
[10] A. Fortin, H. Raybaud-Diogene, B. Tetu, R. Deschenes, J. Huot, J. Landry, Overexpression of the 27 KDa heat shock protein is associated with thermoresistance and chemoresistance but not with radioresistance, International journal of radiation oncology, biology, physics, 46 (2000) 1259-1266.
[11] H.J. Oh, X. Chen, J.R. Subjeck, Hsp110 protects heat-denatured proteins and confers cellular thermoresistance, The Journal of biological chemistry, 272 (1997) 31636-31640.

[12] X. Zhang, Y. Li, Q. Huang, H. Wang, B. Yan, M.W. Dewhirst, C.Y. Li, Increased resistance of tumor cells to hyperthermia mediated by integrin-linked kinase, Clinical cancer research : an official journal of the American Association for Cancer Research, 9 (2003) 1155-1160.
[13] P. Lu, X.Q. Zhu, Z.L. Xu, Q. Zhou, J. Zhang, F. Wu, Increased infiltration of activated tumor-infiltrating lymphocytes after high intensity focused ultrasound ablation of human breast cancer, Surgery, 145 (2009) 286-293.
[14] Z. Yu, J. Geng, M. Zhang, Y. Zhou, Q. Fan, J. Chen, Treatment of osteosarcoma with microwave thermal ablation to induce immunogenic cell death, Oncotarget, 5 (2014) 6526-6539.
[15] A. Zerbini, M. Pilli, A. Penna, G. Pelosi, C. Schianchi, A. Molinari, S. Schivazappa, C. Zibera, F.F. Fagnoni, C. Ferrari, G. Missale, Radiofrequency thermal ablation of hepatocellular carcinoma liver nodules can activate and enhance tumor-specific T-cell responses, Cancer research, 66 (2006) 1139-1146.
[16] S.A. Dromi, M.P. Walsh, S. Herby, B. Traughber, J. Xie, K.V. Sharma, K.P. Sekhar, A. Luk, D.J. Liewehr, M.R. Dreher, T.J. Fry, B.J. Wood, Radiofrequency ablation induces antigen-presenting cell infiltration and amplification of weak tumor-induced immunity, Radiology, 251 (2009) 58-66.
[17] P. Zhou, P. Liang, B. Dong, X. Yu, Z. Han, Y. Xu, Phase clinical study of combination therapy with microwave ablation and cellular immunotherapy in hepatocellular carcinoma, Cancer biology & therapy, 11 (2011) 450-456.
[18] F. Wu, Z.B. Wang, P. Lu, Z.L. Xu, W.Z. Chen, H. Zhu, C.B. Jin, Activated anti-tumor immunity in cancer patients after high intensity focused ultrasound ablation, Ultrasound in medicine & biology, 30 (2004) 1217-1222.
[19] B.W. Dong, J. Zhang, P. Liang, X.L. Yu, L. Su, D.J. Yu, X.L. Ji, G. Yu, Sequential pathological and immunologic analysis of percutaneous microwave coagulation therapy of hepatocellular carcinoma, International journal of hyperthermia : the official journal of European Society for Hyperthermic Oncology, North American Hyperthermia Group, 19 (2003) 119-133.
[20] Z. Hu, X.Y. Yang, Y. Liu, M.A. Morse, H.K. Lyerly, T.M. Clay, P. Zhong, Release of endogenous danger signals from HIFU-treated tumor cells and their stimulatory effects on APCs, Biochemical and biophysical research communications, 335 (2005) 124-131.
[21] G. Schueller, P. Paolini, J. Friedl, A. Stift, P. Dubsky, T. Bachleitner-Hofmann, R. Jakesz, M. Gnant, Heat treatment of hepatocellular carcinoma cells: increased levels of heat shock proteins 70 and 90 correlate with cellular necrosis, Anticancer research, 21 (2001) 295-300.
[22] G. Schueller, J. Kettenbach, R. Sedivy, A. Stift, J. Friedl, M. Gnant, J. Lammer, Heat shock protein expression induced by percutaneous radiofrequency ablation of hepatocellular carcinoma in vivo, International journal of oncology, 24 (2004) 609-613.
[23] J. Nakayama, A. Urabe, H. Terao, S. Taniguchi, Y. Hori, In situ detection of immunocompetent cells in murine B16 melanoma locally treated with interleukin-2 or microwaval hyperthermia, Pigment cell research, 6 (1993) 111-116.
[24] C. Napoletano, F. Taurino, M. Biffoni, A. De Majo, G. Coscarella, F. Bellati, H. Rahimi, S. Pauselli, I. Pellicciotta, J.M. Burchell, L.A. Gaspari, L. Ercoli, P. Rossi, A. Rughetti, RFA strongly modulates the immune system and anti-tumor immune responses in metastatic liver patients, International journal of oncology, 32 (2008) 481-490.
[25] M.Y. Ali, C.F. Grimm, M. Ritter, L. Mohr, H.P. Allgaier, R. Weth, W.O. Bocher, K. Endrulat, H.E. Blum, M. Geissler, Activation of dendritic cells by local ablation of hepatocellular carcinoma, Journal of hepatology, 43 (2005) 817-822.
[26] J.P. Erinjeri, C.T. Thomas, A. Samoilia, M. Fleisher, M. Gonen, C.T. Sofocleous, R.H. Thornton, R.H. Siegelbaum, A.M. Covey, L.A. Brody, W. Alago, Jr., M. Maybody, K.T. Brown, G.I. Getrajdman, S.B. Solomon, Image-guided thermal ablation of tumors increases the plasma level of interleukin-6 and interleukin-10, Journal of vascular and interventional radiology : JVIR, 24 (2013) 1105-1112.
[27] M.C. Jansen, S. van Wanrooy, R. van Hillegersberg, A.M. Rijken, F. van Coevorden, W. Prevoo, T.M. van Gulik, Assessment of systemic inflammatory response (SIR) in patients undergoing radiofrequency ablation or partial liver resection for liver tumors, European journal of surgical oncology : the journal of the European Society of Surgical Oncology and the British Association of Surgical Oncology, 34 (2008) 662-667.
[28] R. Kallio, R. Sequeiros, H.M. Surcel, P. Ohtonen, H. Kiviniemi, H. Syrjala, Early cytokine responses after percutaneous magnetic resonance imaging guided laser thermoablation of malignant liver tumors, Cytokine, 34 (2006) 278-283.

[29] Z. Hu, X.Y. Yang, Y. Liu, G.N. Sankin, E.C. Pua, M.A. Morse, H.K. Lyerly, T.M. Clay, P. Zhong, Investigation of HIFU-induced anti-tumor immunity in a murine tumor model, Journal of translational medicine, 5 (2007) 34.

Thermoresistance			
**Protein symbol**	**TM Index**	**100% accuracy range**	**Estimated Melting Temperature (℃)**
ABCA1	0,790761219	55℃~65	63
ABCB1	0,8397	55-65	64
ABCB8	0,59	55-65	60
ABCC4	0,8801	55-65	64
ABCF2	0,8198	55-65	64
ABCG2	0,6886	55-65	62
BAG1	0,7904	55-65	63
BAG3	1,3227	>65	68
CCNA1	1,7758	>65	72
CDK8	1,4254	>65	69
CRYAA	2,0951	>65	75
CRYGN	1,4721	>65	69
CRYL1	1,5067	>65	70
CRYZL1	1,6749	>65	67
DNAJA1	2,2314	>65	77
DNAJB1	0,0561	55-65	55
DNAJB5	2,9076	>65	84
DNAJB6	1,4748	>65	69
DNAJC15	1,042	>65	65
DNAJC3	1,2607	>65	67
HSF1	1,9936	>65	74
HSP90AA1	0,6188	55-65	61
HSP90AB1	1,1635	>65	66
HSPA12B	0,9433	55-65	65
HSPA1A	1,2719	>65	67
HSPA4	1,7797	>65	72
HSPA6	1,024	>65	65
HSPB1	0,6616	55-65	61
HSPD1	1,791988034	>65	72
HSPH1	1,1335	>65	66
ILK	1,5915	>65	70
PLK1	0,6488	55-65	61
RB1	0,9353	55-65	64

DAMPs/Immunogenic cell death			
**Protein symbol**	**TM Index**	**100% accuracy range**	**Estimated Melting Temperature (°C)**
B3GAT1	-0,1747	<55	53
CALR	-0,8216	<55	47
CD4	1,30091	>65	68
CD68	0,846	55-65	63
CD80	0,828	55-65	63
CD86	1,6222	>65	71
CD8A	1,2732	>65	67
HMGB1	1,5873	>65	70
HSP90AA1	0,6188	55-65	61
HSPA4	1,7797	>65	72
ICAM1	1,3884	>65	68
IFNG	2,2082	>65	77
IL1B	-0,5442	<55	50
IL10	1,3101	>65	68
IL12B	0,2283	55-65	57
IL2RA	0,2359	55-65	57
IL6	0,693566365	55-65	61
ITGAX	1,5517	>65	70
MS4A1	1,3128	>65	68
MUC1	1,5306	>65	70
NCAM1	0,3275	55-65	58
SELL	-0,7243	<55	48
TNF	2,3462	>65	78
TNFRSF1A	0,26588	55-65	57
